# Farnesyl Transferase Inhibitor Lonafarnib Enhances α7nAChR Expression Through Inhibiting DNA Methylation of CHRNA7 and Increases α7nAChR Membrane Trafficking

**DOI:** 10.3389/fphar.2020.589780

**Published:** 2020-12-29

**Authors:** Tingting Chen, Chengyun Cai, Lifeng Wang, Shixin Li, Ling Chen

**Affiliations:** ^1^ Department of Pharmacology, School of Pharmacy, Nantong University, Nantong, China; ^2^ Jiangsu Province Key Laboratory of Inflammation and Molecular Drug Target, Nantong, China; ^3^ School of Life Science, Nantong University, Nantong, China; ^4^ Department of Physiology, Nanjing Medical University, Nanjing, China

**Keywords:** lonafarnib, α7 nicotinic acetylcholine receptor, DNA methylation, c-Jun, c-Jun N-terminal kinase, membrane trafficking

## Abstract

Inhibition of Ras farnesylation in acute has been found to upregulate the α7 nicotinic acetylcholine receptor (α7nAChR) activity. This study was carried out to investigate the effect of chronic administration for 7 days of farnesyl transferase inhibitor lonafarnib (50 mg/kg, intraperitoneally injected) to male mice on the expression and activity of α7nAChR in hippocampal CA1 pyramidal cells. Herein, we show that lonafarnib dose dependently enhances the amplitude of ACh-evoked inward currents (*I*_ACh_), owning to the increased α7nAChR expression and membrane trafficking. Lonafarnib inhibited phosphorylation of c-Jun and JNK, which was related to DNA methylation. In addition, reduced DNA methyltransferase 1 (DNMT1) expression was observed in lonafarnib-treated mice, which was reversed by JNK activator. Lonafarnib-upregulated expression of α7nAChR was mimicked by DNMT inhibitor, and repressed by JNK activator. However, only inhibited DNA methylation did not affect *I*_ACh_, and the JNK activator partially decreased the lonafarnib-upregulated *I*_ACh_. On the other hand, lonafarnib also increased the membrane expression of α7nAChR, which was partially inhibited by JNK activator or CaMKII inhibitor, without changes in the α7nAChR phosphorylation. CaMKII inhibitor had no effect on the expression of α7nAChR. Lonafarnib-enhanced spatial memory of mice was also partially blocked by JNK activator or CaMKII inhibitor. These results suggest that Ras inhibition increases α7nAChR expression through depressed DNA methylation of *CHRNA7 via* Ras-c-Jun-JNK pathway, increases the membrane expression of α7nAChR resulting in part from the enhanced CaMKII pathway and total expression of this receptor, and consequently enhances the spatial memory.

## Introduction

Statins as inhibitors of *de novo* cholesterol biosynthesis can also prevent the production of isoprenoids (Mans et al., 2010), including farnesyl-pyrophosphate (FPP) and geranylgeranyl-pyrophosphate (GGPP). Evidence has indicated that the statins can increase the expression of α7 nicotinic acetylcholine receptor (α7nAChR) in neuroblastoma cells (Roensch et al., 2007), SH-SY5Y cells, and PC12 cells (Xiu et al., 2005). Our previous studies have established that statins can enhance the α7nAChR expression under chronic administration (Chen et al., 2016a) through reducing FPP.

α7nAChR is a ligand-gated ion channel and widely distributed in the central nervous system (e.g., the cerebral cortex and hippocampus). Especially, α7nAChR is highly expressed in the cognition-relevant regions, including the CA1, CA3, dentate gyrus of the hippocampus, and layers I and VI of the cortex; thus, it plays an important role in the memory formation (Hogg et al., 2003; Gotti et al., 2006). More recently, α7nAChR has been found to have a high expression in the interneurons of cortical and pyramidal cells in the hippocampus (Deardorff et al., 2015). α7nAChR can regulate the plasticity of neural circuit, neuronal diﬀerentiation, proliferation, apoptosis, and clearance of aged neurons (Orr-Urtreger et al., 2000). α7nAChR dysfunction plays an important role in the pathogenesis of Alzheimer’s disease (AD). Studies have shown that the α7nAChR expression in the brain changes with age. Interestingly, α7nAChR expression substantially decreases in AD patients (Lykhmus et al., 2015). The disordered expression of *CHRNA7*, the gene encoding α7nAChR, is associated with neuropsychiatric disorders (Gyure et al., 2001; Deutsch et al., 2016). Previous study reported that the cognitive deficits deteriorated in the APP-α7 KO animals when α7nAChR was absent, and the decreased α7nAChRs expression was associated with synaptic damage in AD patients (Pchitskaya et al., 2018); upregulation of α7nAChR expression was able to improve the cognitive deficits (Ma and Qian, 2019).

DNA methylation is an important epigenetic control over different functional genes of the genome (Razin and Cedar, 1991). Canastar et al. (2012) first reported that DNA methylation in the promoter of *CHRNA7* was related to the *CHRNA7* mRNA expression in human cells from various tissue types. They found that SH-EP1 cells with high methylation had no *CHRNA7* expression, and the treatment with methylation inhibitor, that is, 5-aza-2-deoxycytidine, reversed the *CHRNA7* gene silencing in SH-EP1 cells; another methylation inhibitor, that is, zebularine, increased the *CHRNA7* mRNA expression in SH-EP1 and HeLa cells. An inverse correlation between DNA methylation and *CHRNA7* expression has also been reported in human temporal cortical tissues (Dyrvig et al., 2019). This indicates that DNA methylation is crucial for the transcriptional regulation of human *CHRNA7* gene.

DNA methylation is catalyzed by methyltransferases, which are responsible for the formation of 5-methylcytosine (5-mC) from cytosine in the 5′-CpG-3′ dinucleotide (Nowak et al., 2017; Dan et al., 2019). It is catalyzed by DNA methyltransferases (DNMT) and can lead to the mitotic propagation of the modified sequence, resulting in the binding of regulatory proteins such as transcription factors (Calvanese et al., 2009). In mammalian cells, DNMT includes two important classes, DNMT1 and DNMT3 (DNMT3A, DNMT3B, and DNMT3L) (Calvanese et al., 2009; Ions et al., 2013). Different from DNMT3 (Kaneda et al., 2004; Dodge et al., 2005; Drini et al., 2011), DNMT1 is the main type of mammalian DNMT and responsible to maintain the methylation patterns in daughter cells. DNMT1 can be found in almost all somatic cells, but it is highly expressed in proliferating cells (Gruenbaum et al., 1982; Flynn et al., 1996). DNMT3A and -3B are involved in the *de novo* methylation, and highly expressed in embryonic stem cells and early embryos (Kaneda et al., 2004; Dodge et al., 2005); DNMT3L lacks the methyltransferase catalytic domain (Aapola et al., 2001) and is thought to facilitate the action of others (Jia et al., 2007). Rouleau et al. (1995) and MacLeod et al. (1995) found, in Y1 cells derived from a naturally occurring adrenocortical tumor in LAF1 mice, Ras-activator protein 1 (AP-1) (c-Jun) pathway could regulate the activity of DNMT, then influencing DNA methylation (Rouleau et al., 1995). AP-1 is a transcription factor composed of homo- and/or heterodimers of Jun and Fos proteins (Li et al., 2019). The phosphorylation of AP-1 can induce their activation, and then they have transcriptional activity into the nucleus, and c-Jun is activated through phosphorylation by the c-Jun N-terminal kinase (JNK) (Gupta et al., 1996; Kallunki et al., 1996). A recent study reported that hyperactivated Ras can mediate the elevation of phosphorylated-JNK in imaginal discs (Ray and Lakhotia, 2019).

The FPP and GGPP are lipid attachments for the small GTPase (i.e., Ras, Rho, and Rab) superfamily, regulating their prenylation to lead the activation (McTaggart, 2006). The Ras superfamily (e.g., H-Ras, K-Ras, and N-Ras) is a group of representative farnesylated proteins (Kho et al., 2004; McTaggart, 2006), and its functional activity requires farnesylation in the case of FPP, which is catalyzed by the rate-limiting enzyme farnesyl transferase (FTase). Numerous studies have revealed the involvement of Ras signaling pathway in the synaptic plasticity and memory formation (Manabe et al., 2000; Ye and Carew, 2010). Mans’s study and our previous study have reported that reducing FPP (but not GGPP) could upregulate the α7nAChR-dependent long-term potentiation (LTP) (Mans et al., 2010; Chen et al., 2016a) and learning memory, and acute inhibition of Ras farnesylation also could enhance the α7nAChR activity (Chen et al., 2018).

In this study, the FTase inhibitor (FTI) lonafarnib was used to inhibit Ras activation by blocking its farnesylation, and the effects of chronic administration of lonafarnib on the activity and expression of α7nAChR in hippocampal CA1 pyramidal cells were investigated. Our results indicated that chronic Ras inhibition by lonafarnib enhanced α7nAChR expression through inhibiting DNA methylation of *CHRNA7*, which was due to the reduction of DNMT1 *via* Ras-c-Jun-JNK pathway; and increased the membrane expression of α7nAChR, which was mediated in part by CaMKII pathway and enhanced total expression of this receptor, and consequently enhanced the spatial memory of mice.

## Materials and Methods

### Experimental Animals

The present study was approved by the Animal Care and Ethical Committee of Nantong University and Nanjing Medical University. All animal-handling procedures followed the guidelines of Institute for Laboratory Animal Research of the Nantong University and Nanjing Medical University. The procedures involving animals and their care were conducted in conformity with the ARRIVE guidelines of Laboratory Animal Care (Kilkenny et al., 2012). Postnatal 28- to 32-day male mice (C57BL/6J mice, SLAC Laboratory Animal Co., Ltd. Shanghai, China) were maintained in a constant environmental condition (temperature, 23 ± 2°C; humidity, 55 ± 5%; 12:12 h light/dark cycle) in the Animal Research Center of Nantong University and Nanjing Medical University. Animals were given *ad libitum* access to food and water.

### Drug Administration

Ftase inhibitor lonafarnib was purchased from MedChem Express (MCE, NJ, United States). For in *vivo* experiment, lonafarnib was dissolved in DMSO, which then diluted in saline containing 20% (2-hydroxypropyl)-beta-cyclodextrin (Chaponis et al., 2011). Lonafarnib was intraperitoneally injected at different doses of 10, 30, 50, and 80 mg/kg (Chen et al., 2016b) for 7 days. Control mice were intraperitoneally treated with an equal volume of vehicle.

Trans, trans-farnesol (FOH, 96%), and geranylgeraniol (GGOH, 85%) were purchased from Sigma (St. Louis, MO, United States). For in *vivo* experiment, FOH was mixed with 5% Tween 80 to produce an emulsion 30 min prior to lonafarnib administration, and then intraperitoneally injected at 50 mg/kg once daily (de Oliveira Junior et al., 2013). Control mice were intraperitoneally treated with an equal volume of vehicle.

The α7nAChR agonist acetylcholine (ACh) and α7nAChR antagonist methyl lycaconitine (MLA) were purchased from Sigma. The drugs were dissolved in DMSO and diluted by ACSF to a final 0.1% concentration of DMSO, and applied in patch-clamp recording.

CaMKII inhibitor KN93 and DNMT inhibitor RG108 were purchased from MCE. They were dissolved in DMSO, diluted with normal saline, and then injected intracerebroventricularly. Control mice were treated with an equal volume of vehicle. JNK activator anisomycin was purchased from MCE and was dissolved in HCl (1 M), and the concentration was diluted with normal saline to 22 μg/μl; the pH value was adjusted to 7.4 with NaOH (5 M). The vehicle was 1 M HC1 in saline, which was adjusted to pH 7.4 with NaOH.

For repeated intracerebroventricular (i.c.v.) injection of KN-93 (1 μg/5 μl/mouse) and RG108 (20 nmol/5 μl/mouse) (Dong et al., 2019) and anisomycin (110 μg/5 μl/mouse) (Kameyama et al., 1986; Stennett et al., 1989; Robinson and Franklin, 2007), a 28-G stainless steel guide cannula (Plastics One, Roanoke, VA) was implanted into the right lateral ventricle (0.3 mm posterior, 1.0 mm lateral, and 2.5 mm ventral to bregma) and anchored to the skull with three stainless steel screws and dental cement (Wang et al., 2015).

### Electrophysiological Analysis

#### Preparation of Hippocampal Slices

Slice preparation and whole cell patch clamping were performed as previously described (Chen et al., 2018). The mice were anesthetized with isoflurane and sacrificed. Then, the skulls were removed rapidly and sliced with a vibrating microtome (Microslicer DTK 1500, Dousaka EM Co, Kyoto, Japan) in ice-cold cutting solution (in mM: 94 sucrose, 30 NaCl, 4.5 KCl,1 MgCl_2_, 26 NaHCO_3_, 1.2 NaH_2_PO_4_, and 10 D-glucose). The solution was oxygenated with a gas mixture (95% O_2_/5% CO_2_), and the pH value was adjusted to 7.4. The hippocampal slices were then incubated in artificial cerebrospinal fluid (ACSF) containing different concentrations of compounds (in mM: 126 NaCl, 1 CaCl2, 2.5 KCl, 1 MgCl_2_, 26 NaHCO_3_, 1.25 KH_2_PO_4_, and 20 D-glucose, pH 7.4), which was oxygenated with a gas mixture (95% O_2_/5% CO_2_) at 32–34°C using an in-line heating device (Warner Instruments, Hamden, CT).

#### Whole Cell Patch-Clamp Recording

After 1-h recovery in the incubating ACSF, the brain slices were transferred to a recording chamber for whole cell patch-clamp recording. During this process, the slice was continually perfused with oxygenated ACSF. To block muscarinic acetylcholine receptors, atropine (0.5 μM) was added to the external solution. In addition, 10 μM bicuculline, 20 μM AP-5, 10 μM NBQX, and 0.1 μM TTX were applied extracellularly. The patch-clamp recording was performed in pyramidal cells of the hippocampal CA1 region using IR-DIC optics (BX51WI with a ×20 water immersion objective lens, Olympus). Access resistance was monitored continuously during the recording, and the obtained data were discarded if the access resistance fluctuated more than 20%. The glass pipette (4–5 MΩ resistance) was filled with an internal solution (in mM: 120 Cs-gluconate, 2 NaCl, 4 MgCl_2_, 4 Na_2_-ATP, 10 HEPES, and 10 EGTA) at pH 7.2. The holding potential was −70 mV. The a7nAChR-activated current (ACh-evoked inward currents, *I*_ACh_) was induced by adding ACh (0.1–5 mM) *via* a rapid drug delivery system to make sure of the direct application of ACh on the recorded neurons (Colon-Saez and Yakel, 2011; Li et al., 2013). *I*_ACh_ was recorded using an EPC-10 amplifier (HEKA Elektronik, Lambrecht/Pfalz, Germany) and analyzed using pCLAMP 10 software (Molecular Devices), Origin (OriginLab Corp., Northampton, MA, USA), and Sigmaplot10, including peak currents, decay kinetics, and curve fitting. Concerning the varying cell or membrane size, we use the current density, current intensity (pA)/membrane capacitance (pF), to represent the amplitude of *I*_ACh_. *I*_ACh_ (pA/pF) recording from different groups was normalized to *I*_ACh_ (pA/pF) evoked by 5 mM ACh in control to produce dose–response curve. The data were fitted to logistic equation in which *I* = *I*max/[1+(EC50/C)^*n*^], with *n* being Hill coefficient and EC50 being the concentration producing 50% maximal response. α7nAChRs desensitization was calculated by the half-time of desensitization that was required for 50% decay of peak *I*_ACh_ amplitude (Gay et al., 2008).

### Slice Biotinylation and Cell Surface Protein Extraction

Hippocampal slices were placed on a six-well plate and washed with frozen ACSF for 5 min. Then, the hippocampal slices were incubated with ACSF containing EZ-link Sulfo-NHS-SS-Biotin (0.5 mg/ml, Pierce, Northumberland, United Kingdom) for 25 min at 4°C. These slices were washed with ACSF containing 50 mM NH_4_Cl thrice (5 min for each) at 4°C to remove excess biotin. After biotinylation, the hippocampal CA1 region was isolated and homogenized with lysis buffer (50 mM Tris–HCl [pH 7.4], 150 mM NaCl, 1.5 mM MgCl_2_, 1 mM EGTA, 0.5 mM DTT, 50 mM NaF, 2 mM sodium pyruvate, 25% glycerol, 1% triton X-100, 0.5% sodium deoxycholate, and 1% protease inhibitor cocktail, Sigma). The supernatant was centrifuged at 20,000×g for 20 min at 4°C. The resultant supernatant was collected, and the protein concentration was determined by Bradford protein assay. The biotinylated proteins (50 mg) were incubated with streptavidin-coated magnetic beads (30 ml) for 45 min at room temperature. The streptavidin beads containing biotinylated proteins were washed thrice with lysis buffer containing 0.1% SDS and separated with a magnet. The biotinylated proteins were eluted in a sample buffer (62.5 mM Tris–HCl, 2% SDS, 5% glycerol, 5% 2-mercaptoethanol) at 100°C for 5 min. The protein lysates were denatured with the same method. Then, the protein lysates (cytoplasmic proteins) and biotinylated proteins (cell surface proteins) were stored at −20°C until analysis.

### Immunoprecipitation and Western Blotting

Animals were anesthetized, and the brain was harvested, followed by the separation of the hippocampus. The hippocampal tissues or brain slices were homogenized in the lysis buffer containing 50 mM Tris–HCl (pH 7.5), 150 mM NaCl, 5 mM EDTA, 10 mM NaF, 1 mM sodium orthovanadate, 1% Triton X-100, 0.5% sodium deoxycholate, 1 mM phenylmethylsulfonyl fluoride, and protease inhibitor cocktail (Complete; Roche, Mannheim, Germany), followed by incubation for 30 min at 4°C. After cracking with the ultrasonic pulverizer, the samples were centrifuged at 12,000 rpm for 15 min at 4°C, and the supernatant was harvested. The protein concentration was determined with BCA Protein Assay Kit (Pierce Biotechnology Inc., Rockford, IL, United States). Then, equal amount of proteins was mixed with loading buffer and heated in boiling water for 5 min.

For immunoprecipitation assays, total proteins (500 μg) were incubated with rabbit anti-α7nAChR antibody (1:1,000; Chemicon, CA, United States) overnight at 4°C. Then, 40 μg of protein A/G agarose gel (GE Healthcare, Sweden) was added, followed by incubation at 4°C for 1 h. The obtained immunocomplexes were centrifuged at 4°C for 5 min at 1,000 g and washed four times with homogenization buffer (Contreras-Vallejos et al., 2014). The supernatants were collected and subjected to Western blotting.

Equal amount of protein (20 μg) was separated by SDS-polyacrylamide gel electrophoresis (SDS–PAGE), and then transferred onto polyvinylidene fluoride (PVDF) membrane, which was subsequently incubated with blocking solution (5% nonfat milk) for 60 min at room temperature. After washing thrice, the membrane was incubated overnight at 4°C with following antibodies: rabbit polyclonal anti-α7nAChR (1:1,000; Abcam, Cambridge, United Kingdom), DNMT1 (1:1,000; Abcam, Cambridge, MA, USA), DNMT3A (1:1,000; Abcam), DNMT3B (1:1,000; Abcam), anti-phospho-c-Jun (1:1,000; Millipore, MA, United States), anti-phospho-JNK (1:1,000; Millipore, MA, United States), anti-phospho-Ser (1:1,000; Santa Cruz, CA, United States), anti-phospho-Thr (1:1,000; Santa Cruz, CA, United States), anti-phospho-PKA (1:1,000; Millipore, MA, United States), anti-phospho-PKC (1:1,000; Abcam, Cambridge, United Kingdom), and anti-phospho-CaMKII (1:1,000; Cell Signaling Technology, MA, United States). After washing, the membrane was incubated with HRP-labeled secondary antibodies at room temperature for 1–2 h, and visualization was done with the ECL Detection Kit (Millipore, MA, USA). The blots were stripped by incubation in stripping buffer (Restore, Pierce) for 5 min, blocked with 5% nonfat milk at room temperature for 60 min, and then incubated with anti-c-Jun, anti-JNK (1:1,000; Millipore), anti-PKC (1:1,000; Abcam), anti-PKA (1:1,000; Millipore), and anti-CaMKII (1:1,000; Abcam). Internal control was GAPDH or β-actin (1:2000; Cell Signaling Technology). The biotinylated membrane surface a7nAChR protein was normalized by surface GluR2 protein (John et al., 2015; Chen et al., 2018). We used two methods of exposure, traditional darkroom exposure or using the exposure machine, for α7nAChR and phospho-CaMKII/CaMKII, and the traditional one seemed more suitable. ImageJ (NIH Image, Bethesda, MD, USA) was used to determine the protein expression which was normalized to the expression of internal control.

### Reverse Transcription–Polymerase Chain Reaction (RT-PCR)

Real-time RT–PCR was performed as described previously (Albers et al., 2014). Total RNA was isolated from the hippocampus with TRIzol reagent (Invitrogen, Camarillo, CA) and reverse-transcribed into cDNA using a Prime Script RT Reagent Kit (Takara, China) for quantitative PCR (ABI Step One Plus, Foster City, CA) in the presence of fluorescent dye (SYBR Green I; Takara, China). The relative expression of genes was determined using the 2^−ΔΔct^ method with *GAPDH* as an internal control. The primers used for PCR were as follows: *DNMT1*, 5′-CGT​TGT​GGT​GGA​TGA​CAA​GA-3′ and 5′-GAA​CCA​GGA​CAG​TGG​CTC​T-3′; *DNMT3A*; 5′-GTG​CAG​AAA​CAT​CGA​GGA​CA-3′ and 5′-ATG​CCT​CCA​ATG​AAG​AGT​GG-3′; *DNMT3B*, 5′-ACA​ACC​GTC​CAT​TCT​TCT​GG-3′ and 5′-GTG​AGC​AGC​AGA​CAC​CTT​GA-3′ (Miozzo et al., 2018); *α7nAChR*, 5′-CAC​ATT​CCA​CAC​CAA​CGT​CTT-3′ and 5′-AAA​AGG​GAA​CCA​GCG​TAC​ATC-3′ (Albers et al., 2014); *α4nAChR*, 5′-CAG​CTT​CCA​GTG​TCA​GAC​CA-3′ and 5′-TGG​AAG​ATG​TGG​GTG​ACT​GA-3′ (Ghedini et al., 2012); *β2nAChR*, 5′-GAG​GTG​AAG​CAC​TTC​CCA​TTT-3′ and 5′-GCC​ACA​TCG​CTT​TTG​AGC​AC-3′ (Albers et al., 2014); and *GAPDH*, 5′-TGG​GTG​TGA​ACC​ACG​AG-3′ and 5′-AAG​TTG​TCA​TGG​ATG​ACC​TT-3′.

### Morris Water Maze (MWM)

Morris water maze test was conducted for 8 consecutive days to detect the spatial cognitive function of mice (Tong et al., 2012). Morris water maze consists of a circular pool (made of black plastic and 120 cm in diameter) that is artificially divided into four quadrants and marked on the wall with entry points for each quadrant. The water temperature was maintained at 23 ± 2°C, and an appropriate amount of white food additives was added into the water. Swim paths were analyzed using a computer system with a video camera (AXIS-90 Target/2; Neuroscience). During the experiment, the reference outside the maze should remain unchanged, and the indoor environment should be kept quiet to avoid interfering with the experiment. In the first two days of training (visible platform test), a cylindrical dark-colored platform (7 cm in diameter) was placed 0.5 cm above the surface of water. During training days 3–7 (hidden platform test), the acquisition testing phase, the platform was submerged 1 cm below the water surface. Mice were given 90 s in the pool to search the platform. Latency to reach the visible or the hidden platform, and the swim distance were measured. During the training, if the mouse failed to find the platform within 90 s, it would be guided to the platform, and the trial was terminated. Each mouse started from one of the four quadrants randomly, with its head toward the wall. Four trials were conducted every day with an interval of 30 min. On day 8, the retention of spatial reference memory was recorded by a probe trial with the platform being removed from the pool, and the percent time spent in each quadrant was assessed.

### Data and Statistical Analysis

All statistical analyses were performed with GraphPad Prism 8. Data are presented as mean ± s. e.m unless stated otherwise. Differences among means were analyzed using Student’s *t*-test and analyses of variance (ANOVA) with or without repeated measures, followed by *post hoc* analysis. *P*- and *F*-values of ANOVAs are given in the results or figure legends section. Differences at *P* < 0.05 were considered statistically significant. Repeats for experiments and statistical tests carried out are indicated in the figure legends and in the main text, respectively.

## Results

### Effects of Lonafarnib on α7nAChR Activity in Hippocampal CA1 Pyramidal Cells

**FIGURE1:**
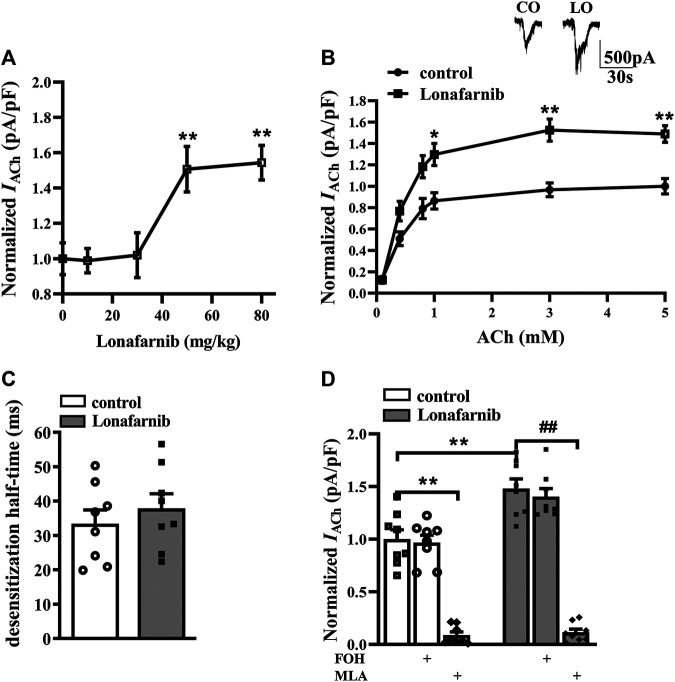
Administration of lonafarnib enhances a7nAChR activity in hippocampal CA1 pyramidal cells. **(A)** Evoked *I*_ACh_ by ACh (3 mM) in the slices of control mice treated with vehicle and mice treated with lonafarnib at 10, 30, 50, and 80 mg/kg for 7 days. Dose–response curves were constructed by the amplitude of *I*_ACh_ (the value of current density as pA/pF) (means ± SEM) that expressed as percent of control with vehicle (100% VS control). **P < 0.01 vs. slices of control mice (10 mg/kg: *P* = 0.9999, 30 mg/kg: *P* = 0.9998; 50 mg/kg: *P* = 0.0062; 80 mg/kg: *P* = 0.0032, *n* = 8, one-way ANOVA, followed by Dunnett’s multiple comparison test). **(B)** The CA1 pyramidal cells were subjected to consecutive 1 s applications of 0.1, 0.4, 0.8, 1, 3, and 5 mM ACh in control and lonafarnib-treated mice (50 mg/kg). Dose–response curves were constructed by the amplitude of *I*_ACh_ (means ± SEM) that were normalized by a control value evoked by ACh (5 mM). Representative traces of *I*_ACh_ evoked by 3 mM ACh. *P < 0.05, **P < 0.01 vs. slices of control mice (1 mM: *P* = 0.0298, 3 mM: *P* = 0.0043, 5 mM: *P* = 0.0025, *n* = 8, repeated-measure ANOVA, followed by Sidak’s multiple comparison test). **(C)** Influence of lonafarnib administration (50 mg/kg) on the desensitization half-time of a7nAChR. **(D)** Sensitivity of evoked *I*_ACh_ to farnesol (FOH) that converts farnesyl-pyrophosphate and a7nAChR antagonist MLA. Mean percent reduction in the amplitude of *I*_ACh_ evoked by ACh (3 mM) following application of MLA (10 μM) in slices from control and lonafarnib-treated mice. Amplitude of *I*_ACh_ were normalized by the value of control group with vehicle. **P < 0.01 vs. slices of control mice; ^**##**^ *P* < 0.01 vs. slices of lonafarnib-treated mice (two-way ANOVA, followed by Tukey’s multiple comparison test).

To investigate the effect of lonafarnib on the α7nAChR activity, the ACh-evoked inward current (*I*_ACh_) was recorded in mouse brain slices after 7 days of lonafarnib treatment at different doses of 10, 30, 50, and 80 mg/kg (Chen et al., 2016b). *I*_ACh_ of hippocampal CA1 pyramidal cells was examined by whole cell patch-clamp recording. Compared with control mice, the administration of lonafarnib increased the amplitude of *I*_ACh_ (pA/pF) in a dose-dependent manner (*F*_4,35_ = 7.393, *P* = 0.0002, one-way ANOVA; Figure 1A). According to this result, the influence of lonafarnib (50 mg/kg) treatment for 7 days on the sensitivity of α7nAChR to agonists was further investigated. ACh dose–response curves were delineated to evaluate the response of α7nAChR to different concentrations of ACh (Figure 1B). As shown in the dose–response curves, a concentration-dependent increase of *I*_ACh_ amplitude was observed in both groups (*F*_5,70_ = 83.94, *P* < 0.0001, repeated measure ANOVA). Lonafarnib significantly increased the *I*_ACh_ amplitude compared to control mice (*F*_1,14_ = 23.28, *P* = 0.0003, repeated measure ANOVA; Figure 1B). In addition, EC50 and Hill coefficient were comparable between Lonafarnib-treated mice (EC50 = 407.4 μM; Hill coefficient = 1.864) and control mice (EC50 = 430.7 μM; Hill coefficient = 2.021). Lonafarnib had no influence on the desensitization half-time (ms) of *I*_ACh_ (*t*-test as t 14) = 0.7632, *P* = 0.4580; Figure 1C). Then, lonafarnib at 50 mg/kg and ACh at 3 mM were used in the following experiments. In addition, α7nAChR antagonist MLA (10 μM) was also used to confirm that the recorded current and upregulation by lonafarnib were attributed to α7nAChR, but not other cholinergic receptor. As shown in Figure 1D, *I*_ACh_ was sensitive to MLA (vs. control mice or lonafarnib-treated mice: *P* < 0.0001, *n* = 8, two-way ANOVA with Tukey’s test; Figure 1D) in 5 min in both groups. However, mice treated with FOH had no influence in the currents (*vs* control mice: *P* = 0.9998, or lonafarnib-treated mice: *P* = 0.9742, *n* = 8, two-way ANOVA, followed by Tukey’s multiple comparison test; Figure 1D). These results showed that lonafarnib (50 mg/kg) treatment for 7 days potentiated the a7nAChR activity, without changing the agonist sensitivity and the kinetics of desensitization.

### Influence of Lonafarnib on the Expression of Hippocampal nAChR

**FIGURE 2:**
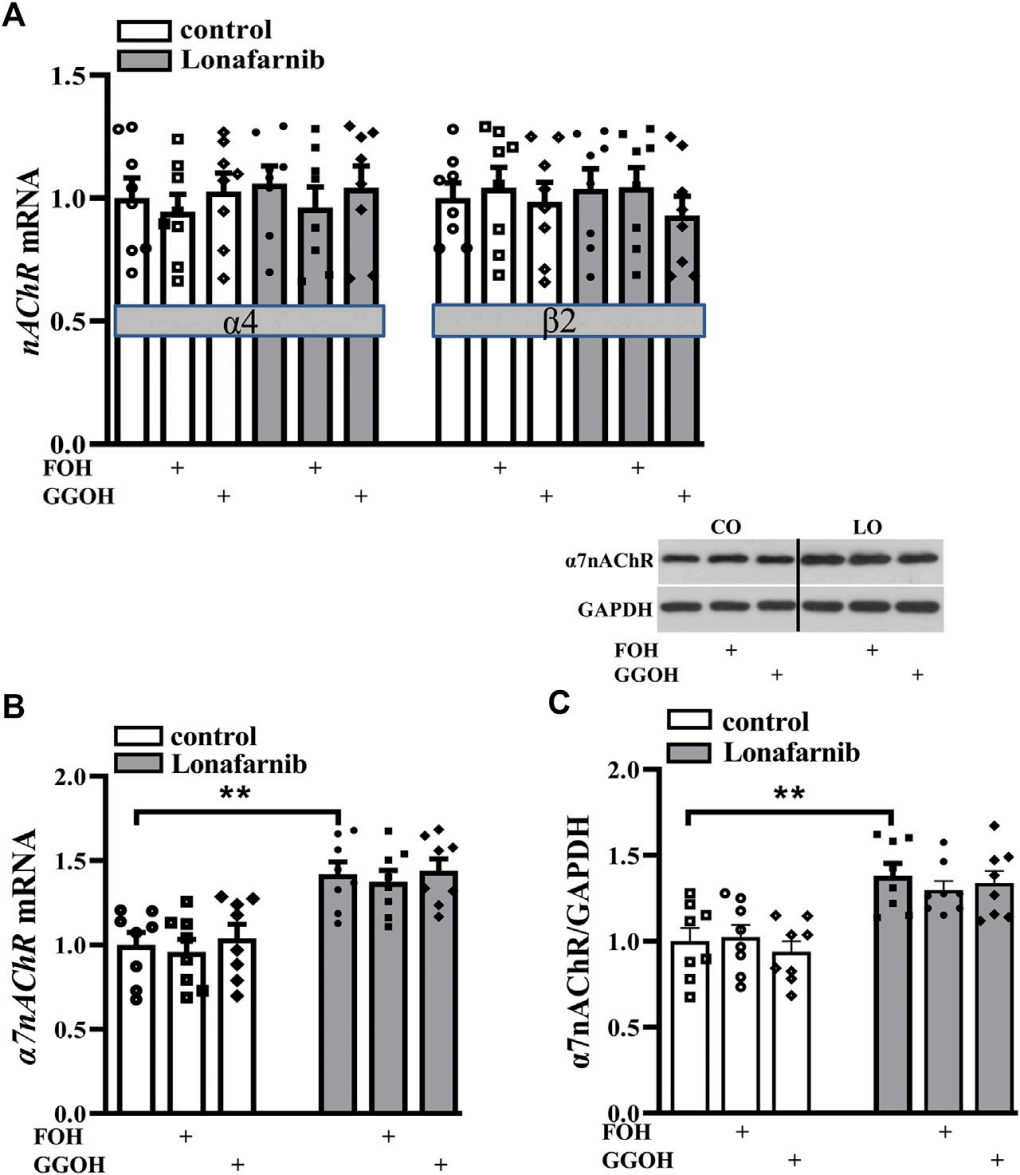
Lonafarnib administration increases the expression of hippocampal α7nAChR, but not α4/β2nAChR. **(A)** Levels of *α4/β2nAChR* mRNA in the hippocampus of control mice and lonafarnib-treated mice administrated with vehicle, FOH, or GGOH. **(B)** Level of *α7nAChR* mRNA in the hippocampus of control mice and lonafarnib-treated mice treated with vehicle, FOH, or GGOH. **P < 0.01 vs. control mice (two-way ANOVA, followed by Tukey’s multiple comparison test). **(C)** Level of α7nAChR protein in the hippocampus. **P < 0.01 vs. control mice (two-way ANOVA, followed by Tukey’s multiple comparison test). CO: control mice, LO: lonafarnib-treated mice. The expression of mRNA and protein were normalized by the values of control group with vehicle.

Among the various forms of nAChRs, only two subtypes are highly expressed in the central nervous system, α7-subunit containing homomer (α7nAChR) and α4β2 heteromer (α4β2nAChR) (Ma and Qian, 2019). To investigate the effect of lonafarnib (50 mg/kg) on the expression of α7nAChR and α4β2nAChR, RT-PCR and Western blotting were employed to detect the mRNA and protein expression, respectively. Our results showed the mRNA expression of α4/β2nAChR was similar between lonafarnib-treated mice and control mice (*P* > 0.9999, *n* = 8, two-way ANOVA, followed by Tukey’s multiple comparison test; Figure 2A). By contrast, α7nAChR mRNA expression (*P* = 0.0019, *n* = 8; two-way ANOVA, followed by Tukey’s multiple comparisons test; Figure 2B) and α7nAChR protein expression (*P* < 0.0011, *n* = 8; Figure 2C) in the lonafarnib-treated mice increased significantly as compared to control mice. Moreover, the enhanced mRNA and protein expression of α7nAChR were not affected by FOH (50 mg/kg) (mRNA: *P* = 0.9991, *n* = 8; protein: *P* = 0.6837, *n* = 8) or GGOH (50 mg/kg) (mRNA: P > 0.9999, *n* = 8; protein: *P* = 0.9789, *n* = 8), which can be metabolized into FPP or GGPP (Chen et al., 2016a). These results indicated that the lonafarnib-induced upregulation of α7nAChR expression may be connected to gene transcription, for not only the a7 protein increased but also the *a7* mRNA (Xiu et al., 2005). Lonafarnib could completely inhibit the farnesylation of Ras because of no influence of FOH or GGOH.

### Effect of Lonafarnib on DNA Methylation of CHRNA7

Studies have reported the correlation between *CHRNA7* (a gene encoding α7nAChR) mRNA and DNA methylation in the promoter of *CHRNA7* in various types of tissue (Razin and Cedar, 1977; Razin and Shemer, 1999; Canastar et al., 2012; Dyrvig et al., 2019). Activation of c-Jun has been reported to result in hyper-induction of DNMT promoter, then influencing DNA methylation (Rouleau et al., 1995). To explore the mechanism underlying the lonafarnib-induced enhancement of α7nAChR expression, the c-Jun-JNK pathway was examined, and the expression of DNMT was detected in the presence of lonafarnib treatment.

**FIGURE 3:**
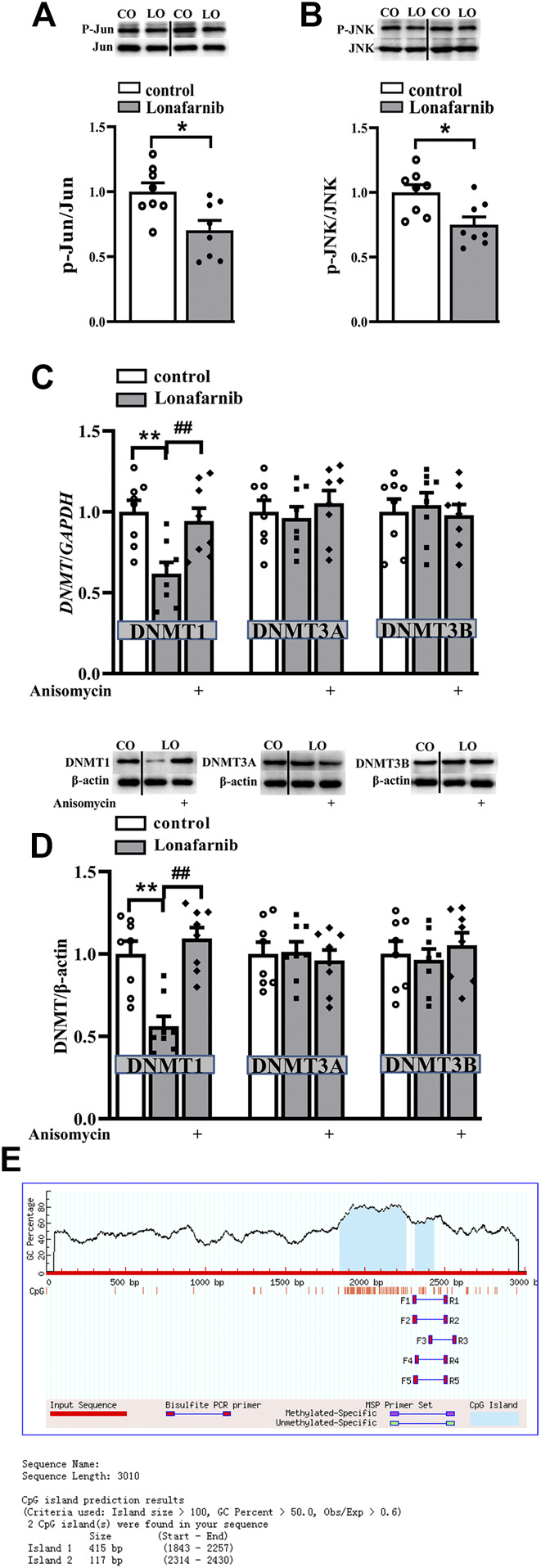
Lonafarnib administration downregulates the phosphorylation of C-Jun and JNK pathway and, in this way, decreases DNMT1 in the hippocampus. **(A and B)** Levels of phospho-Jun and phospho-JNK in control and lonafarnib-treated mice, *P < 0.05 vs. control mice (*t*-test). **(C)** Levels of *DNMT1/3A/3B* mRNA in the hippocampus of control mice and lonafarnib-treated mice treated with vehicle or anisomycin. **P < 0.01 vs. control mice; ^**##**^ *P* < 0.01 vs. lonafarnib-treated mice (two-way ANOVA, followed by Tukey’s multiple comparison test). **(D)** Levels of DNMT1/3A/3B proteins in the hippocampus of control mice and lonafarnib-treated mice treated with vehicle or anisomycin, **P < 0.01 vs. control mice; ^**##**^ *P* < 0.01 vs. lonafarnib-treated mice (two-way ANOVA, followed by Tukey’s multiple comparison test). **(E)** Prediction of CpG island in the promoter region of *CHRNA7* gene. The expression of mRNA and protein were normalized by the values of control group with vehicle.

Our results showed significantly decreased phosphorylation of c-Jun (phospho-c-Jun) and JNK (phospho-JNK) in the lonafarnib-treated mice as compared to control mice (phospho-c-Jun: *t*-test as t 14) = 2.910, *P* = 0.0114; phospho-JNK: t-test as t 14) = 2.960, *P* = 0.0103; Figures 3A,B). Meanwhile, the protein and mRNA expression of DNMT1, DNMT3A, and DNMT3B were detected. Results showed lonafarnib reduced the mRNA (*P* = 0.0023, *n* = 8, two-way ANOVA, followed by Tukey’s multiple comparison test; Figure 3C) and protein (*P* = 0.0020, *n* = 8, Figure 3D) expression of DNMT1, which both could be reversed by anisomycin, an activator of JNK (mRNA: *P* = 0.0078, *n* = 8; protein: *P* = 0.0004, *n* = 8; Figures 3C,D). However, the mRNA (*DNMT3A*: *P* = 0.7928, *n* = 8; *DNMT3B*: *P* = 0.9092, *n* = 8) and protein (DNMT3A: *P* = 0.9570, *n* = 8; DNMT3B: *P* = 0.4325, *n* = 8) expressions of DNMT3A and DNMT3B remained unchanged. Few studies have reported the methylation of *CHRNA7* promoter in mice, and we predicted the presence of CpG island in the *CHRNA7* gene promoter by using MethPrimer. As shown in Figure 3E, two CpG islands were found in the promoter region of *CHRNA7* at 1843–2,257 bp and 2,314–2,430 bp, the GC content is more than 50%, and observed CpG to expected CpG dinucleotide ratio (ObsCpG/ExpCpG) was more than 0.60. In addition, previous study has demonstrated the CHRNA7 proximal promoter CpG island in human cells, including SH-EP1, HeLa, SH-SY5Y, and SK-N-BE cells (Canastar et al., 2012). These findings indicated the possibility of the regulation by DNA methylation in the *CHRNA7* promoter in mice, and lonafarnib might decrease DNA methylation through reducing the expression of DNMT1, which was regulated by the c-Jun-JNK pathway.

### The Role of DNA Methylation Alteration in the Lonafarnib Induced Upregulation of α7nAChR, and the Alteration of Membrane Expression and Phosphorylation of α7nAChR in Lonafarnib-Treated Mice

**FIGURE 4:**
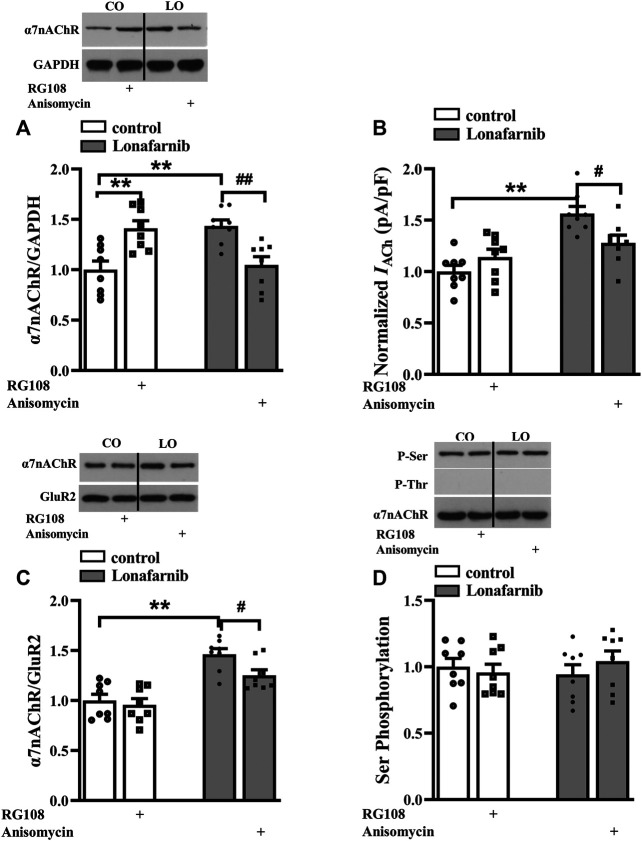
Role of DNA methylation in the lonafarnib affected activity, total expression, membrane expression, and phosphorylation of a7nAChR. **(A)** Levels of a7nAChR total proteins in the hippocampus of control and lonafarnib-treated mice treated with vehicle, DNMT inhibitor RG108 or anisomycin. **P < 0.01 vs. control mice, ^**##**^ *P* < 0.01 vs. lonafarnib-treated mice (two-way ANOVA, followed by Tukey’s multiple comparison test). **(B)** Evoked *I*_ACh_ by ACh (3 mM) in the slices of control and lonafarnib-treated mice treated with vehicle, RG108, or anisomycin, **P < 0.01 vs. control mice; ^**#**^ *P* < 0.05 vs. lonafarnib-treated mice (two-way ANOVA, followed by Tukey’s multiple comparison test). **(C)** Levels of biotinylated a7nAChR (membrane surface) protein in the hippocampus of control and lonafarnib-treated mice treated with vehicle, RG108, or anisomycin. Surface a7nAChR was normalized by surface GluR2 protein, which was again normalized by vehicle-treated control group. **P < 0.01 vs. control mice; ^**#**^ *P* < 0.05 vs. lonafarnib-treated mice (two-way ANOVA, followed by Tukey’s multiple comparison test). **(D)** Levels of phospho-a7nAChR in the hippocampus of control and lonafarnib-treated mice treated with vehicle, RG108, or anisomycin. The expression of protein and the amplitude of evoked *I*_ACh_ were normalized by the values of control group with vehicle.

To explore the role of DNA methylation in the lonafarnib-induced upregulation of α7nAChR expression and activity, mice were treated with JNK activator anisomycin and DNMT inhibitor RG108. Interestingly, results showed RG108 inhibited the DNA methylation in control mice, which mimicked the effect of lonafarnib and could induce an increase of α7nAChR expression (*P* < 0.0001, *n* = 8, two-way ANOVA, followed by Tukey’s multiple comparison test; Figure 4A); however, RG108 slightly upregulated *I*_ACh_ as compared to control mice, with no significant difference (*P* = 0.5197, *n* = 8, two-way ANOVA, followed by Tukey’s multiple comparison test; Figure 4B). Anisomycin significantly inhibited lonafarnib-induced increase of α7nAChR expression (*P* = 0.0001, *n* = 8, Figure 4A) and induced a smaller inhibition of *I*_ACh_ in lonafarnib-treated mice (*P* = 0.0266, *n* = 8, Figure 4B). The different effects of DNA methylation inhibitor and lonafarnib on the expression and activity of α7nAChR suggest other mechanisms underlying the lonafarnib-induced upregulation of α7nAChR activity. As a membrane receptor, the functional expression of α7nAChR is affected by its membrane expression and phosphorylation (Cho et al., 2005; Gillentine et al., 2017). Then, whether lonafarnib also affected the membrane trafficking and phosphorylation of α7nAChR and their relationships with the increased α7nAChR expression were further explored. Results showed lonafarnib significantly upregulated the membrane expression of α7nAChR (*P* < 0.0001, *n* = 8, vs control mice, two-way ANOVA, followed by Tukey’s multiple comparison test; Figure 4C), which was partially inhibited by anisomycin (*P* = 0.0141, *n* = 8, vs lonafarnib-treated mice, Figure 4C). In control mice, DNMT inhibitor RG108 had no significant effect on the membrane expression (*P* = 0.9548, *n* = 8, vs control mice, Figure 4C). However, phosphorylation of α7nAChR remained unchanged in the control and lonafarnib-treated mice (*P* = 0.7695 *n* = 8, two-way ANOVA, followed by Tukey’s multiple comparison test; Figure 4D).

### Pathways Related to the Upregulated Membrane Expression in Lonafarnib-Treated Mice

**FIGURE 5:**
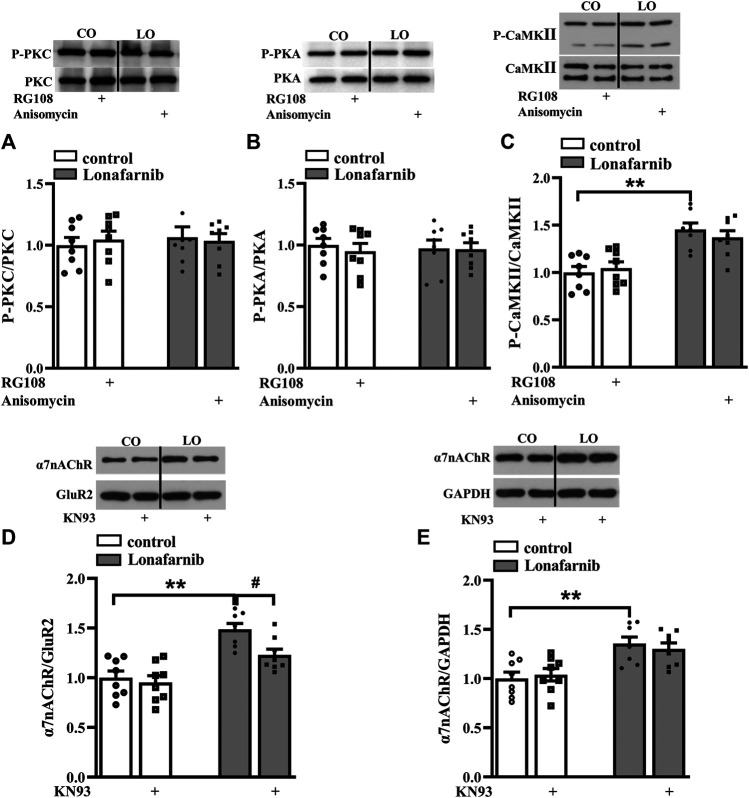
Lonafarnib administration affects CaMKII signaling pathways, partially modulating the membrane expression of a7nAChR. **(A and B)** Levels of phospho-PKC and phospho-PKA in the hippocampus of control and lonafarnib-treated mice treated with vehicle, RG108, or anisomycin. **(C)** Levels of phospho-CaMKII in the hippocampus of control and lonafarnib-treated mice treated with vehicle, RG108, or anisomycin. **P < 0.01 vs. control mice (two-way ANOVA, followed by Tukey’s multiple comparison test). **(D)** Levels of biotinylated a7nAChR (membrane) protein in the hippocampus of control and lonafarnib-treated mice treated with vehicle or CaMKII pathway blocker KN93. Surface a7nAChR was normalized by surface GluR2 protein, which was again normalized by vehicle-treated control group. **P < 0.01 vs. control mice; ^**#**^ *P* < 0.05 vs. lonafarnib-treated mice (two-way ANOVA, followed by Tukey’s multiple comparison test). **(E)** Levels of a7nAChR total proteins in the hippocampus of control and lonafarnib-treated mice treated with vehicle and KN93, and total a7nAChR was normalized by GAPDH, which was again normalized by vehicle-treated group. **P < 0.01 vs. control mice (two-way ANOVA, followed by Tukey’s multiple comparison test). The expression of protein was normalized by the values of control group with vehicle.

A large body of evidence indicates that the activation of small GTPases may alter their interactions with intracellular molecules to regulate downstream effectors including PKC, PKA, and CaMKII (McTaggart, 2006). The membrane trafficking of α7nAChR is regulated by PKC and CaMKII signaling pathways (Komal et al., 2015), and the phosphorylation of α7nAChR is modulated by PKA and PKC (Huganir and Greengard, 1990; Moss et al., 1996). Our previous study showed that acute perfusion with statins and FTI-277 could upregulate the membrane trafficking of α7nAChR through CaMKII or PKC pathway (Chen et al., 2018). Therefore, we further explore whether chronic administration with lonafarnib also affected these pathways to upregulate the membrane expression of α7nAChR. The effects of lonafarnib, RG108, and anisomycin on the phosphorylation of PKCε (phospho-PKCε), CaMKII (phospho-CaMKII), and PKA (phospho-PKA) were examined. Notably, lonafarnib elevated the expression of phospho-CaMKII (*P* < 0.001, *n* = 8, two-way ANOVA, followed by Tukey’s multiple comparison test; Figure 5C), but not phospho-PKCε (*P* = 0.8099, *n* = 8, two-way ANOVA, followed by Tukey’s multiple comparison test; Figure 5A) or phospho-PKA (*P* = 0.9615, *n* = 8, two-way ANOVA, followed by Tukey’s multiple comparison test; Figure 5B). Neither RG108 in control mice nor anisomycin in lonafarnib-treated mice had influence on the expression of phospho-CaMKII (RG108: *P* = 0.9597, *n* = 8; anisomycin: *P* = 0.7383, *n* = 8), phospho-PKCε (RG108: *P* = 0.9366, *n* = 8; anisomycin: *P* = 0.9885, *n* = 8), or phospho-PKA (RG108: *P* = 0.7382, *n* = 8; anisomycin: *P* = 0.9998, *n* = 8 Figures 5A–C). Furthermore, lonafarnib-induced increase of membrane expression could be partially inhibited by KN93, an inhibitor of CaMKII pathway (*P* = 0.0119, *n* = 8, two-way ANOVA, followed by Tukey’s multiple comparison test; Figure 5D). However, the increased total expression in lonafarnib-treated mice was not altered by KN93 (*P* = 0.7528, *n* = 8, two-way ANOVA, followed by Tukey’s multiple comparison test; Figure 5E). These results indicated that lonafarnib stimulated membrane trafficking of α7nAChR partially through CaMKII pathway, but not PKC or PKA pathway, and the DNA methylation had no influence in the pathways. Besides, upregulated CaMKII pathway had no effect on the total expression of α7nAChR.

### The Influence of Lonafarnib in the Spatial Memory

**FIGURE 6:**
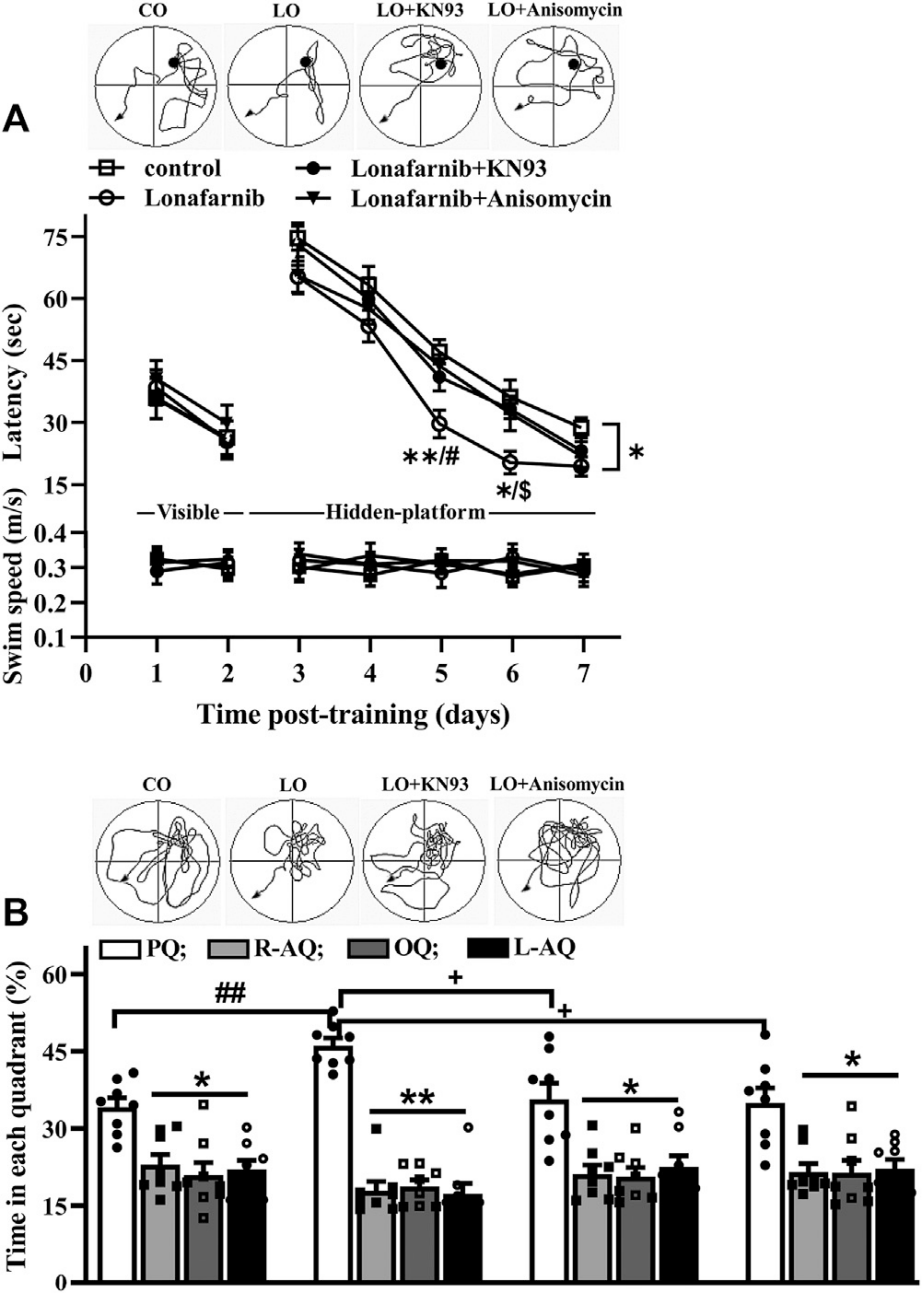
Lonafarnib treatment improves spatial learning memory. **(A)** Latency (sec) to reach visible platform and hidden platform of Morris water maze in control mice (control), lonafarnib-treated mice (lonafarnib), lonafarnib + KN93-treated mice (lonafarnib + KN93), and lonafarnib + anisomycin-treated mice (lonafarnib + anisomycin). Tracings of typical swim patterns in hidden platform task (upper panels). Black circles indicate the position of platform. *P < 0.05, and **P < 0.01 vs. control mice, ^#^ *P* < 0.05 vs. Lonafarnib + anisomycin-treated mice, ^$^ *P* < 0.05 vs. lonafarnib + KN93-treated mice (repeated-measure ANOVA, followed by Tukey’s multiple comparison test). **(B)** Percentage of swim time (%) in quadrants of platform (PQ), opposite (OQ) and right/left adjacent (R-AQ, L-AQ) in Morris water maze. Tracings of typical swim patterns in probe task (upper panels). **P < 0.01 and *P < 0.05 vs. swim time in PQ (comparison within group, repeated-measure ANOVA, followed by Dunnett’s multiple comparison test), ^##^ *P* < 0.01 vs. control mice, ^+^ *P* < 0.05 vs. lonafarnib + KN93-treated mice or + anisomycin-treated mice (one-way ANOVA, followed by Tukey’s multiple comparison test).

Then, we carried out behavior test by Morris water maze to examine the influence of lonafarnib in the spatial memory, and the involvement of α7nAChR. In the MWM test, the latency in visible platform can reflect the search behavior or visual acuity; latency in hidden platform is used to judge the spatial learning and memory. As shown in Figure 6A (upper), the latency to reach the visible platform was affected by training days (*F*_1,28_ = 182.0, *P* < 0.0001, repeated measure ANOVA; Figure 6A); however, there is no difference between four groups (*F*_3,28_ = 0.2525, *P* = 0.8589). The escape latency to reach the hidden platform was progressively decreased with training days in four groups (*F*_4,112_ = 211.2, *p* < 0.0001). Repeated measures ANOVA revealed a difference between four groups in the latency to reach the hidden platform (*F*_3,28_ = 3.390, *P* = 0.0317, followed by Tukey's multiple comparison test), and the latency of lonafarnib-treated mice was reduced compared with control mice (*P* = 0.0358). Mice treated with lonafarnib take less time to reach the hidden platform on days 5–6 after training than the control mice (day 5: *P* = 0.0078; day 6: *P* = 0.0306). Compared with lonafarnib-treated mice, mice treated by lonafarnib + KN93 showed reversed effect on the time to reach the hidden platform at day 6 (*P* = 0.014, *n* = 8), and mice with lonafarnib + anisomycin treatment presented increased time to reach hidden platform at day 5 (*P* = 0.0256, *n* = 8). KN93 and anisomycin treatment seemed to have a tendency to reverse the reduced latency in lonafarnib-treated mice; however, no overall significant difference was found in the lonafarnib + KN93-treated mice (*P* = 0.2806, *n* = 8) or + anisomycin mice (*P* = 0.4503, *n* = 8) compared with lonafarnib-treated mice. There was no significant difference in swimming speed during the training days (visible and hidden) between four groups (*F*_6,168_ = 0.2207, *P* = 0.9445, repeated measures ANOVA; Figure. 6A, bottom). A probe trial was performed at 24 h after the hidden platform test, in which the swimming time spent in four quadrants (platform, opposite, right, and left adjacent quadrants) was measured to estimate the memory trace strength. The swimming time spent in the target quadrant was longer than that in other quadrants in control mice (*F*_3,28_ = 6.846, *P* = 0.0033, repeated measures ANOVA, followed by Dunnett's multiple comparisons test; PQ VS R-AQ: *P* = 0.0151; VS OQ: *P* = 0.0196; VS L-AQ: *P* = 0.0101; *n* = 8; Figure 6B). Notably, lonafarnib-treated mice showed more swim time in platform quadrant than in control mice (*P* = 0.0087, *n* = 8, one-way ANOVA, followed by Tukey’s multiple comparison test). The enhancement of swim time in platform quadrant in lonafarnib-treated mice was blocked by the KN93 treatment (*P* = 0.0208, *n* = 8) and anisomycin treatment (*P* = 0.0124, *n* = 8). The results indicate the due spatial memory improvement in lonafarnib-treated mice, which is partially influenced when using KN93 or anisomycin to inhibit the activation of α7nAChR.

**FIGURE 7:**
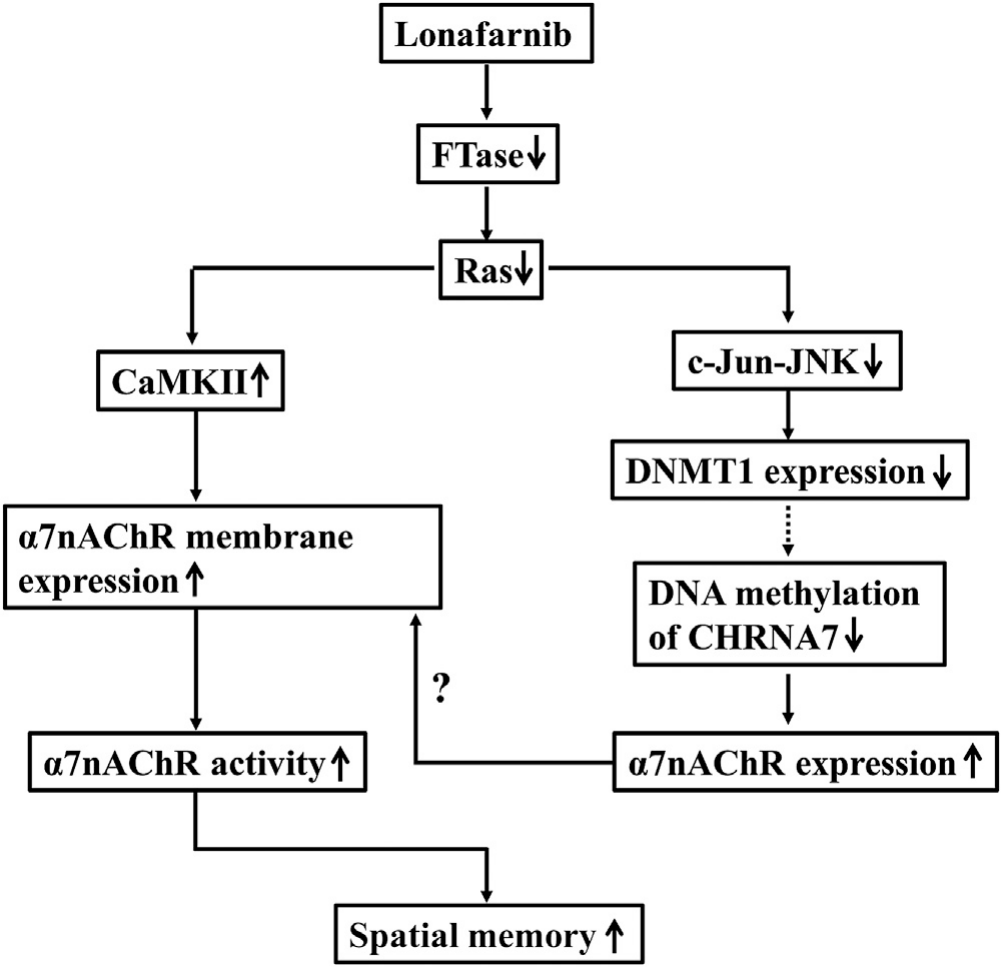
The hypothesis of molecular mechanism underlying the lonafarnib-upregulated α7nAChR. ↑: increase; ↓: decrease; dotted line meant hypothesis need to be confirmed.

## Discussion

To our knowledge, the present study, for the first time, provided evidence that Ras inhibition by lonafarnib could enhance the total expression of α7nAChR through inhibiting DNA methylation of *CHRNA7* promoter by decreasing DNMT1, and increase the membrane trafficking of α7nAChR which was mediated in part by CaMKII pathway and the enhanced total expression, leading to an upregulation of α7nAChR activity in hippocampal CA1 pyramidal cells, and consequently improved the spatial memory of mice.

Homomeric α7 and heterometric α4β2 receptors are most abundant in the nervous system; each subtype has its unique activation, agonist selectivity, channel conductance, and desensitization properties (Houchat et al., 2020). Activation of α4β2nAChRs triggers slowly decaying nicotinic currents (Matsubayashi et al., 2004), while α7 subtype is distinguished by its rapid desensitization (Couturier et al., 1990; Seguela et al., 1993). In addition, a7nAChRs can be fully activated by agonists such as choline, nicotine, and ACh, and inhibited reversibly by MLA and irreversibly by α-bungarotoxin (α-BGT). For α4β2nAChRs, they are activated by ACh and nicotine, but not by choline, inhibited by dihydro-berythroidine (DHβE), but not by a-BGT or MLA (Alkondon and Albuquerque, 2001). As previous study has reported in hippocampal neurons (Alkondon and Albuquerque, 1993), based upon the decay kinetics of the currents elicited by 3 mM ACh, the neurons were shown to exhibit different current types; among them, type I_A_ currents (rapidly decaying currents) were the most frequent and were found in 83% of the neurons tested, mediated by α7nAChR, and completely inhibited by MLA or α-BGT, or κ-BGT alone. 10% of the neurons had mixed responses (named type I_B_), which were mediated by α7nAChR and α4β2nAChR, partially blocked by MLA or DHβE alone, completely blocked by combination of the two agents. α4β2nAChR mediated (type II) currents shown in 5% of neurons, which could be inhibited by DHβE, but not α-BGT. This classification was very similar to that found in nigral neurons as previously described (Matsubayashi et al., 2004). In our experiments, when analyzing the currents mediated by α7nAChR, we chose *I*_ACh_ with typical fast desensitization, and then the chosen ones were also confirmed by the complete inhibition of MLA. The electrophysiological analysis revealed that lonafarnib treatment for 7 days increased the maximal α7nAChR response, without altering the half-time (ms) of α7nAChR desensitization, EC50, and Hill coefficient of the dose–response curve. These findings suggest that lonafarnib treatment enhances the activity of α7nAChR, but not the affinity of α7nAChR. FOH had no effect on the lonafarnib-upregulated *I*_ACh_, which indicates that when the rate-limiting enzyme FTase is inhibited by lonafarnib, supplement of FPP fails to rescue the activation of Ras. This finding was also confirmed by the receptor expression detection. Our results showed lonafarnib treatment for 7 days significantly increased the expression of mRNA and protein of α7nAChR, but failed to affect the expression of α4β2nAChR. Consistent with the electrophysiological results, FOH had no influence on the lonafarnib-induced increase of α7nAChR expression.

In the mammalian genome, DNA methylation is an epigenetic mechanism which can regulate gene expression by recruiting proteins involved in gene repression or by inhibiting the binding of transcription factor(s) to DNA (Moore et al., 2013). A link between promoter DNA methylation and tissue-specific transcription of *CHRNA7* has been reported by Canastar et al. (2012). They found that *CHRNA7* expression was silenced in the SH-EP1 cells with a region corresponding high methylation level. However, in the SH-SY5Y and HeLa cells with demethylated regions, the expression of *CHRNA7* was significantly higher, suggesting the crucial role of DNA methylation in the transcriptional regulation of human *CHRNA7* gene. Moreover, Dyrvig et al. (2019) found that DNA methylation at three promoter regions was involved in the regulation of *CHRNA7* transcription. In our study, a significant reduction of DNMT1 was observed in the lonafarnib-treated mice. RG108, an inhibitor of DNMT, could mimic the effect of lonafarnib on the α7nAChR expression. This finding reveals that lonafarnib-induced enhancement of α7nAChR expression is related to the downregulation of DNA methylation through reducing DNMT1. Patra (2008) reported that Ras could regulate DNMT1 and DNA-methylation, which also interacted with many proteins during cell cycle progression. A 106-bp sequence (at -1744 to -1,650) of *DNMT* gene bearing three AP-l sites was responsible for the induction of *DNMT* promoter activity (Rouleau et al., 1995). In Y1 cells, the activation of *DNMT* promoter is controlled by the Ras-AP-1 (c-Jun) signaling pathway (MacLeod et al., 1995; Rouleau et al., 1995). Expression of a down-modulator of Ras activity, or a trans-dominant negative mutant of Jun in Y1 cells, decreases the mRNA expression, enzymatic activity, and DNA-methylation of *DNMT*. In our study, lonafarnib-induced Ras inactivation reduced the phosphorylation of both c-Jun and JNK, and decreased the DNMT1 expression; furthermore, the reduced DNMT1 expression could be rescued by anisomycin, an activator of JNK. Our results suggest that lonafarnib inhibits Ras activation to decrease DNMT1 expression *via* c-Jun-JNK pathway, which consequently reduces DNA-methylation, resulting in the increase of α7nAChR expression. However, other mechanisms may be also involved in the effect of lonafarnib on the DNA methylation, since the link between DNA methylation and histone markers has been reported (Ooi et al., 2007; Kelly et al., 2010), and inhibition of histone deacetylases (HDACs) induces global DNA hypomethylation (Arzenani et al., 2011). A recent study also showed treatment with HDAC inhibitor Valproate immediately induced effective demethylation and significantly increased the *CHRNA7* promoter gene expression in HeLa cells (Dyrvig et al., 2019). In our previous study, results showed simvastatin could reduce FPP to upregulate the histone acetylation (Chen et al., 2016b). Cellular histone acetylation is maintained by the histone acetyltransferases (HAT) and HDAC (Kramer et al., 2001), and inhibition of HDAC may enhance the histone acetylation. However, little is known about the relationship between Ras signaling and HDAC, which may need further investigation.

Our results showed anisomycin, an activator of JNK, significantly reduced lonafarnib-upregulated expression of α7nAChR, but partially inhibited the current of α7nAChR increased by lonafarnib. Similarly, DNMT inhibitor RG108 increased the expression of α7nAChR, but slightly elevated *I*_ACh_ with no significance. These results remind that other mechanisms may be also involved in the lonafarnib-upregulated activity of α7nAChR besides the increased expression of α7nAChR. As an integral membrane receptor, α7nAChR must be folded and assembled in the endoplasmic reticulum (ER), followed by membrane trafficking of assembled receptors (Gillentine et al., 2017). In this way, the membrane expression of α7nAChR may reflect the activity of α7nAChR (Drisdel et al., 2004; Ma and Qian, 2019). Phosphorylation at serine/threonine also modulates α7nAChRs activity (Huganir and Greengard, 1990). Cho et al. (2005) reported that the amount of functional cell surface α7nAChR was controlled indirectly *via* processes involving phosphorylation. However, in our study, results showed lonafarnib upregulated the membrane expression of α7nAChR, but had no effect on the phosphorylation of α7nAChR. A large body of evidence indicates that activation of small GTPases may alter their interactions with intracellular molecules to regulate downstream effectors including PKC, PKA, and CaMKII (McTaggart, 2006). Although studies have reported the role of PKC, PKA, and CaMKII in the membrane trafficking and phosphorylation of α7nAChR (Moss et al., 1996; Kanno et al., 2012a; Kanno et al., 2012b), our results showed chronic lonafarnib treatment enhanced the phosphorylation of CaMKII, but not PKC or PKA, and lonafarnib upregulated membrane expression was partially inhibited by CaMKII inhibitor KN93. This was consistent with our previous finding that FTase inhibitor (FTI-277) acute incubation for 4 h increased the membrane trafficking of α7nAChR through CaMKII pathway but not PKC or PKA. Interestingly, chronic lonafarnib treatment induced enhancement of membrane trafficking of α7nAChR was also partially reduced by JNK activator anisomycin, which decreased lonafarnib-upregulated α7nAChR expression. This indicates the involvement of increased total expression in the enhanced membrane trafficking in the lonafarnib-treated mice. It has been revealed that long-term treatment with neuregulin may enhance the expression and function of α7nAChR in the hippocampal cells (Liu et al., 2001; Kawai et al., 2002). The normal translocation of α7nAChR from intracellular pools to the surface is clearly an important but not well-defined process (Dineley and Patrick, 2000). Cho et al. (2005) reported that brief exposure of rat hippocampal interneurons to genistein potentiated α7nAChR responses, which was related to the enhanced expression of surface α7 subunits, resulting from the increased expression of α7nAChRs. However, in other cases, they thought that in chromaffin cells, when the total expression was enhanced, most upregulated nAChRs were stored in an intracellular pool, while surface receptors might actually be depressed (Peng et al., 1997; Warpman et al., 1998; Ridley et al., 2001; Sala et al., 2008). Based on our findings, lonafarnib enhanced the total expression of α7nAChR through reducing DNA methylation of *CHRNA7* promoter *via* c-Jun-JNK pathway, and this increased expression was attributed to the enhanced membrane trafficking of the receptor, which was also regulated by the CaMKII pathway. The mechanism underlying the interaction between lonafarnib-enhanced α7nAChR expression and activity is not completely understood and worth further exploring.

α7nAChR is highly expressed in the cognition-relevant regions, playing a crucial role in memory formation (Hogg et al., 2003; Gotti et al., 2006). Cholinesterase inhibitors (ChEIs), which can recover the level of acetylcholine (activator of α7nAChR) in the central nervous system in AD brain, is one of the two types of medications for AD treatment approved by the Food and Drug Administration (Uddin et al., 2020). From our previous study, statins could significantly enhance the spatial cognitive performance as assessed by Morris water maze and Y-maze in adult mice through reducing FPP (Chen et al., 2016a). And the enhancement resulted from statins in synaptic plasticity assessed by LTP, a cellular model of learning and memory (Bliss and Collingridge, 1993), could be mimicked by FTI-277 (Mans et al., 2012). In our study, we due found an enhancement of lonafarnib treatment in the spatial memory as tested by Morris water maze. In the hidden platform test, the latency was significantly changed by lonafarnib treatment, especially on days 5 and 6. This enhancement was decreased to certain extent by KN93 or anisomycin, and mice treated by lonafarnib + KN93 showed an inverse of latency on day 6, and those treated by lonafarnib + anisomycin showed an inverse on day 5; however, both two groups had no significance in latency with lonafarnib-treated mice overall. In the probe trial, lonafarnib-treated mice exhibited an obvious increase in the time spent in platform quadrant than control mice, which was partially inhibited by KN93 or anisomycin. From the results of MWM, we can see that KN93 or anisomycin could not completely block the improvement in the spatial memory induced by lonafarnib. There may be other mechanisms involved in the benefit of spatial memory resulted from lonafarnib treatment. Foregoing results showed that affecting Ras-c-Jun-JNK or CaMKII pathway in part altered α7nAChR activity, which might explain the incomplete inhibition of the two drugs. Moreover, FTI-277 treatment enhanced the activity of NMDA receptor (Chen et al., 2016b), which played a crucial role in the learning and memory. In this way, inhibition of α7nAChR might not totally inverse the enhancement of spatial memory resulted from lonafarnib.

However, these evidences observed in our study do provide a support to the memory benefits of lonafarnib through the activation of α7nAChR, which also can predict the feasibility of Ras inhibitors in the anti-dementia treatment in AD patients.

## Data Availability Statement

The raw data supporting the conclusions of this article will be made available by the authors, without undue reservation.

## Ethics Statement

The animal study was reviewed and approved by Nantong University, Nanjing Medical University.

## Author Contributions

TC performed the electrophysiology recordings, a part of Western Blotting and PCR, all statistical analysis and wrote the manuscript, CC carried out a part of Western Blotting and PCR, LW, and SL carried out the animal care. LC was responsible for the experimental design and manuscript drafting.

## Funding

This study was supported by the National Natural Science Foundation of China (Grant number 81901098), Natural Science Foundation of Jiangsu province (Grant number BK20190923), and Science and technology Project of Nantong City (Grant numbers JC2018009 and JC2019026).

## SUPPLEMENTARY MATERIAL

The Supplementary Material for this article can be found online at: https://www.frontiersin.org/articles/10.3389/fphar.2020.589780/full#supplementary-material.

## Conflict of Interest

The authors declare that the research was conducted in the absence of any commercial or financial relationships that could be construed as a potential conflict of interest.

## Abbreviations

FPP, farnesyl-pyrophosphate; GGPP, geranylgeranyl-pyrophosphate; α7nAChR, α7 nicotinic acetylcholine receptor; AD, Alzheimer’s disease; 5-mC, 5-methylcytosine; DNMT, DNA methyltransferases; AP-1, Activator protein 1; JNK, c-Jun N-terminal kinase; FTase, Farnesyl transferase; LTP, long-term potentiation; FTI, FTase inhibitor; FOH, Farnesol; GGOH, Geranylgeraniol; ACh, Acetylcholine; MLA, Methyl lycaconitine; ACSF, Artificial cerebrospinal fluid; *I*_ACh_, ACh-evoked inward currents; HDACs Histone deacetylases; HAT, Histone acetyltransferases; MWM, Morris water maze; ChEIs, Cholinesterase inhibitors.
