# Supplementary material for: Farnesyl Transferase Inhibitor Lonafarnib Enhances α7nAChR Expression Through Inhibiting DNA Methylation of CHRNA7 and Increases α7nAChR Membrane Trafficking
Source: Front Pharmacol. 2020 Dec 29;11:589780. doi: 10.3389/fphar.2020.589780 (PMC7801264; doi:10.3389/fphar.2020.589780)
Supplement: Supplementary file 3 [file datasheet3.pdf]

Fig 2D

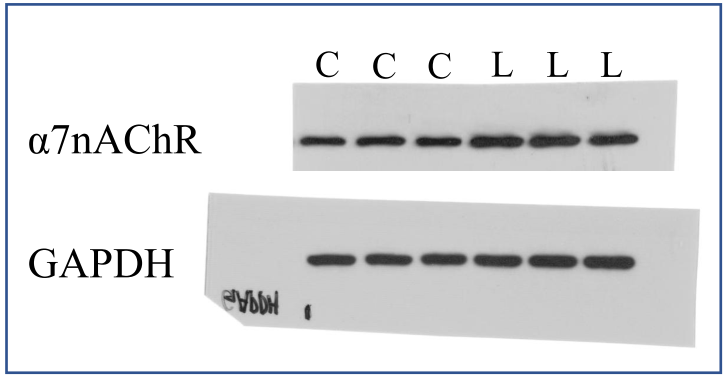

Fig 3A

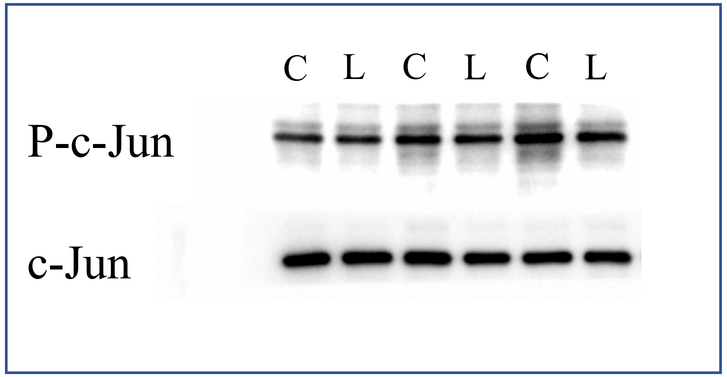

Fig 3B

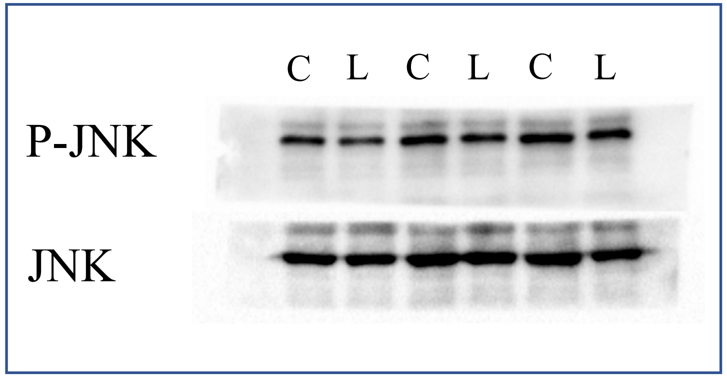

Fig 3D-DNMT1

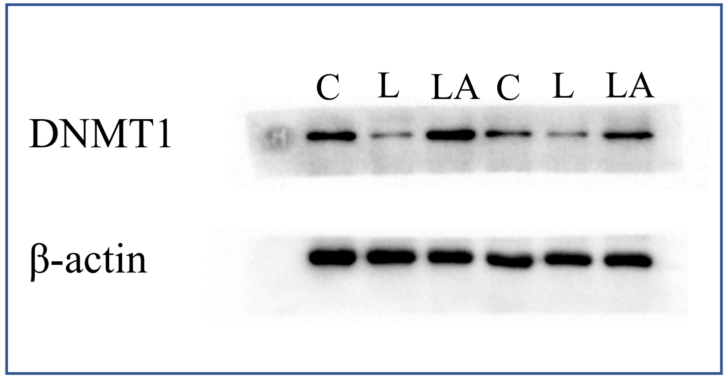

Fig 3D-DNMT3A

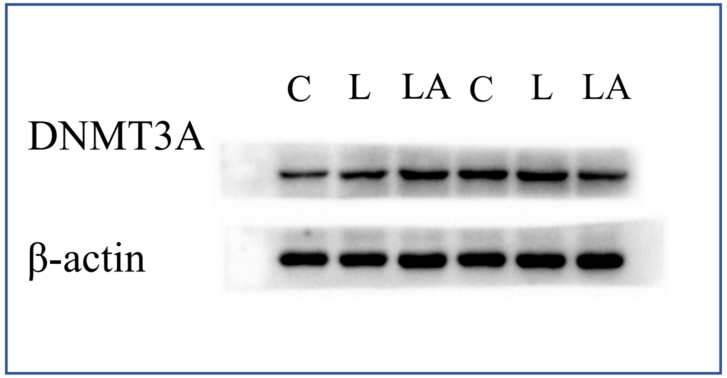

Fig 3D-DNMT3B

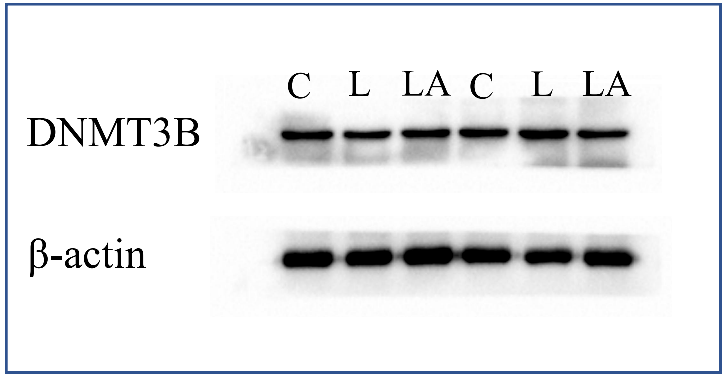

Fig 4A

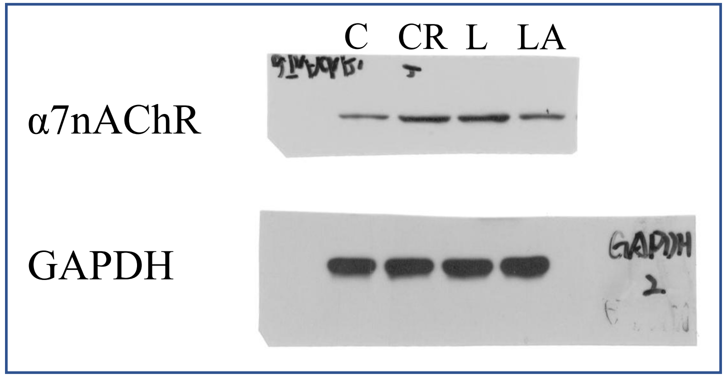

Fig 4C

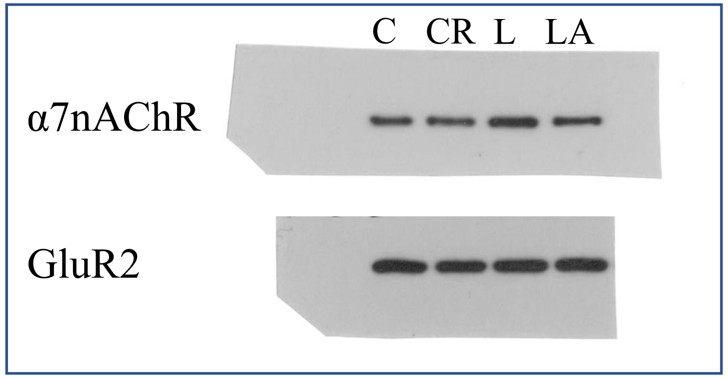

C: Control;
L: Lonafarnib;
CR: Control+RG108;
LA: Lonafarnib+Anisomycin;

Fig 4D

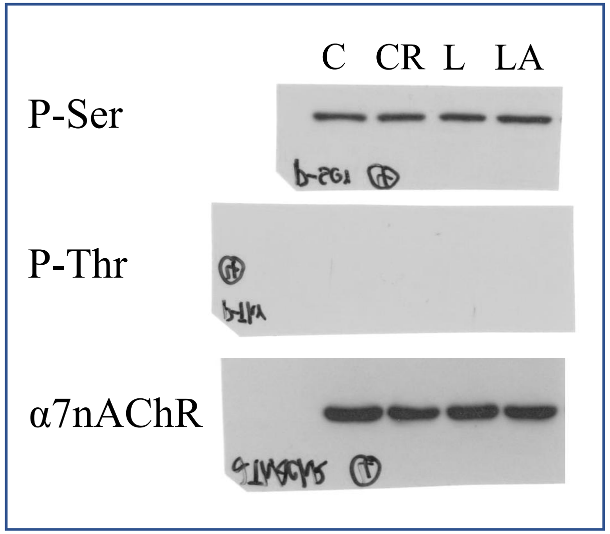

Fig 5A

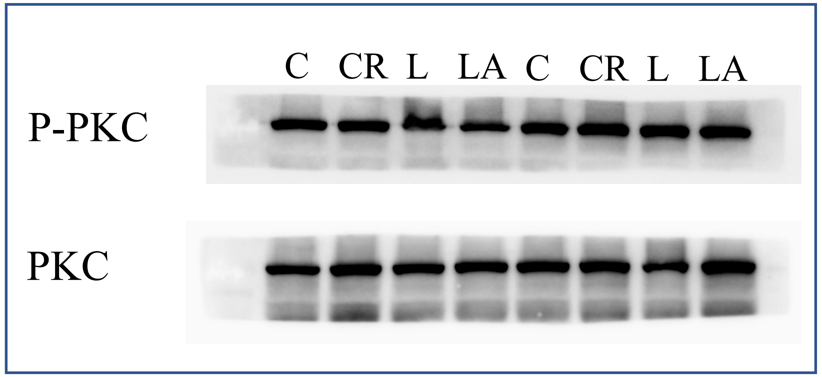

Fig 5B

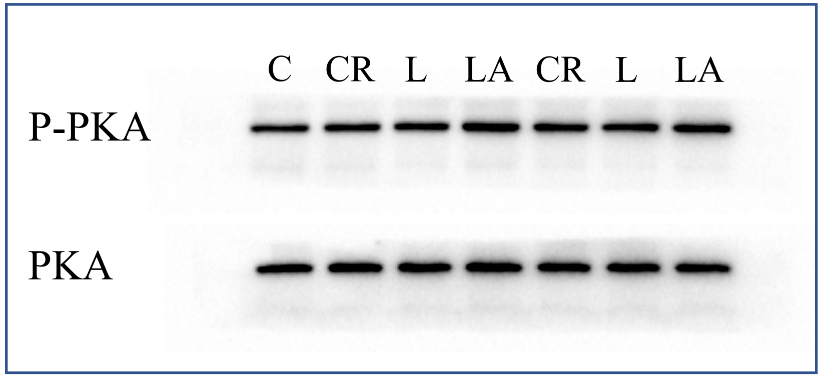

Fig 5C

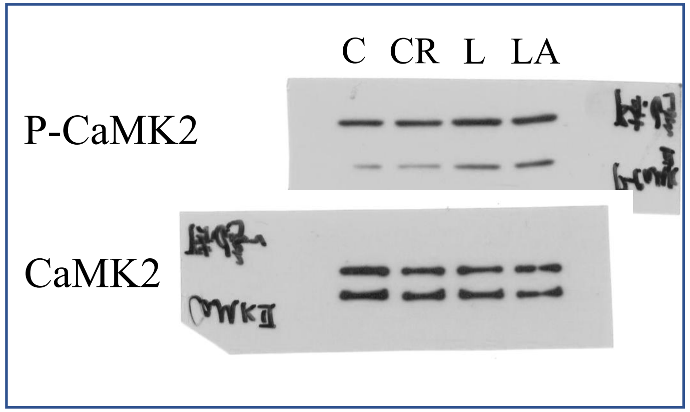

Fig 5D

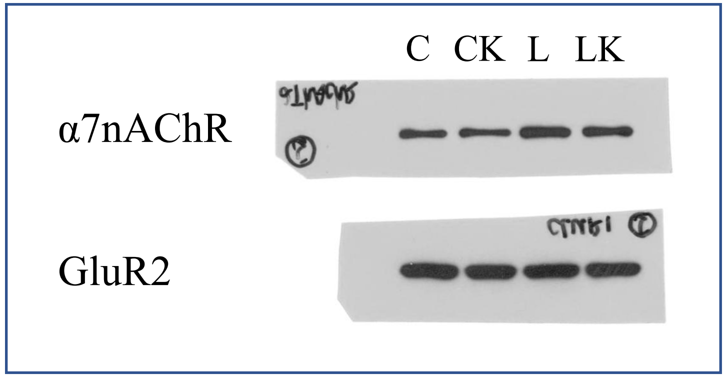

Fig 5E

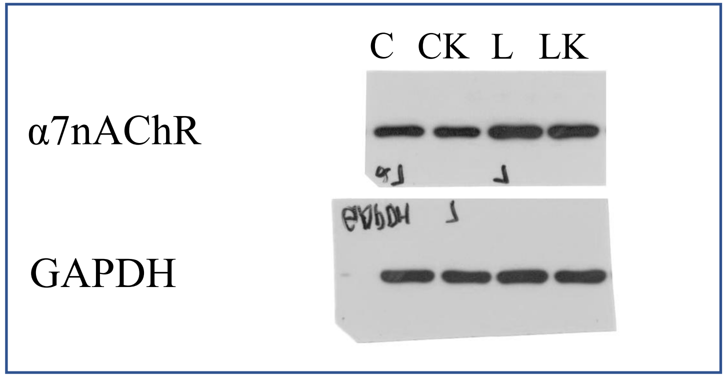

C： Control；
L： Lonafarnib;
CR: Control+RG108;
LA: Lonafarnib+Anisomycin;
CK: Control+KN93;
LK: Lonafarnib+KN93
